# Rebamipide Attenuates Mandibular Condylar Degeneration in a Murine Model of TMJ-OA by Mediating a Chondroprotective Effect and by Downregulating RANKL-Mediated Osteoclastogenesis

**DOI:** 10.1371/journal.pone.0154107

**Published:** 2016-04-28

**Authors:** Takashi Izawa, Hiroki Mori, Tekehiro Shinohara, Akiko Mino-Oka, Islamy Rahma Hutami, Akihiko Iwasa, Eiji Tanaka

**Affiliations:** Department of Orthodontics and Dentofacial Orthopedics, Institute of Biomedical Sciences, Tokushima University Graduate School, Tokushima, Japan; China Medical University, TAIWAN

## Abstract

Temporomandibular joint osteoarthritis (TMJ-OA) is characterized by progressive degradation of cartilage and changes in subchondral bone. It is also one of the most serious subgroups of temporomandibular disorders. Rebamipide is a gastroprotective agent that is currently used for the treatment of gastritis and gastric ulcers. It scavenges reactive oxygen radicals and has exhibited anti-inflammatory potential. The aim of this study was to investigate the impact of rebamipide both *in vivo* and *in vitro* on the development of cartilage degeneration and osteoclast activity in an experimental murine model of TMJ-OA, and to explore its mode of action. Oral administration of rebamipide (0.6 mg/kg and 6 mg/kg) was initiated 24 h after TMJ-OA was induced, and was maintained daily for four weeks. Rebamipide treatment was found to attenuate cartilage degeneration, to reduce the number of apoptotic cells, and to decrease the expression levels of matrix metalloproteinase-13 (MMP-13) and inducible nitric oxide synthase (iNOS) in TMJ-OA cartilage in a dose-dependent manner. Rebamipide also suppressed the activation of transcription factors (e.g., NF-κB, NFATc1) and mitogen-activated protein kinases (MAPK) by receptor activator of nuclear factor kappa-B ligand (RANKL) to inhibit the differentiation of osteoclastic precursors, and disrupted the formation of actin rings in mature osteoclasts. Together, these results demonstrate the inhibitory effects of rebamipide on cartilage degradation in experimentally induced TMJ-OA. Furthermore, suppression of oxidative damage, restoration of extracellular matrix homeostasis of articular chondrocytes, and reduced subchondral bone loss as a result of blocked osteoclast activation suggest that rebamipide is a potential therapeutic strategy for TMJ-OA.

## Introduction

Temporomandibular joint osteoarthritis (TMJ-OA) is a degenerative joint disease that is characterized by the death of chondrocytes, loss of cartilage extracellular matrix (ECM), and subchondral bone resorption in its early stages, followed by abnormal reparative bone turnover [1–4]. Under most conditions, osteoclast-mediated bone resorption and bone formation are tightly coupled. However, when the amount of bone resorption exceeds that of bone formation, subchondral bone loss often occurs [5].

Recent studies have implicated the inflammatory process in the pathogenesis of osteoarthritis (OA) [6]. Moreover, accumulating evidence has shown that cartilage-degrading proteinases and proinflammatory cytokines, such as matrix metalloproteinase-13 (MMP-13) and interleukin (IL)-1β, can promote catabolic processes that lead to the degeneration of cartilage and subchondral bone [7].

Similar to other autoimmune diseases, including rheumatoid arthritis (RA), Sjögren’s syndrome, and Behcet’s disease, oxidative stress is also involved in the pathology of OA [8–10]. Chronic oxidative stress refers to a condition that is characterized by elevated production of reactive oxygen species (ROS). In diseases like OA and RA, deregulation of cellular proliferation and excess nitric oxide (NO) formation are hallmarks of cartilage degradation [11]. Inducible nitric oxide synthase (iNOS) in chondrocytes produces NO in response to IL-1, TNF-α, and LPS [12]. In the presence of high concentrations of NO, chondrocytes then undergo apoptosis [13], and this apoptosis is a commonly accepted hallmark of OA [14,15]. Furthermore, the apoptosis of chondrocytes appears to positively correlate with the severity of matrix depletion and destruction that are observed in osteoarthritic cartilage [15–17].

Rebamipide (2-[4-chlorobenzoylamino]-3-[2(1*H*)quinolinon-4-yl] propionic acid; OPC-12759) is a mucosal protective agent that is currently used for the treatment of gastritis and gastric ulcers that are induced by nonsteroidal anti-inflammatory drugs (NSAIDs). Rebamipide has been shown to act as an oxygen radical scavenger of cytokine-induced hydroxyl radicals [18], and has exhibited anti-inflammatory activity [19]. In rats, rebamipide treatment has been shown to prevent dextran sulfate sodium-induced colitis [20], while recent studies in a murine model of Sjögren’s syndrome demonstrated that rebamipide attenuates inflammatory and apoptotic lesions in the salivary and lacrimal glands [21,22].

Given the anti-oxidant and anti-inflammatory properties that have been observed for rebamipide, the aim of the present study was to investigate the effects of rebamipide on mandibular condylar cartilage deterioration and on various parameters of local oxidative damage and inflammatory responses in a repetitive bite opening-induced TMJ-OA mouse model. We hypothesize that rebamipide will exhibit anti-inflammatory activity in the mandibular condyles of TMJ-OA model mice consistent with a beneficial therapeutic effect.

## Materials and Methods

### Ethics

This study was conducted in accordance with the Fundamental Guidelines for Proper Conduct of Animal Experiments and Related Activities in Academic Research Institutions under the jurisdiction of the Ministry of Education, Culture, Sports, Science and Technology of the Japanese Government. This study was approved by the Ethics Committee of Tokushima University for Animal Research (Approval #: toku-12122 and toku-12134). The mice were anesthetized during all of the experiments and all efforts were made to minimize their suffering. They were euthanized by cervical dislocation after being rendered unconscious from exposure to CO_2_.

During the experimental procedures, each mouse was monitored twice daily for health status. No mice died or were euthanized prematurely due to severe illness or becoming moribund. The early euthanasia/humane endpoint criteria were: loss of > 20% body weight the presence of a wound that does not heal with medication development of signs of neurological abnormality, or an inability to eat independently.

### Mice

Eight-week-old C57BL/6 wild-type (WT) mice were purchased from Japan SLC Inc. (Shizuoka, Japan) and maintained under specific pathogen-free conditions. They were provided with food and water *ad libitum* and housed in a room that was held at a constant ambient temperature (22–24°C) with a 12-h light/12-h dark cycle.

### TMJ-OA model induction and rebamipide treatment

**Fig 1:**
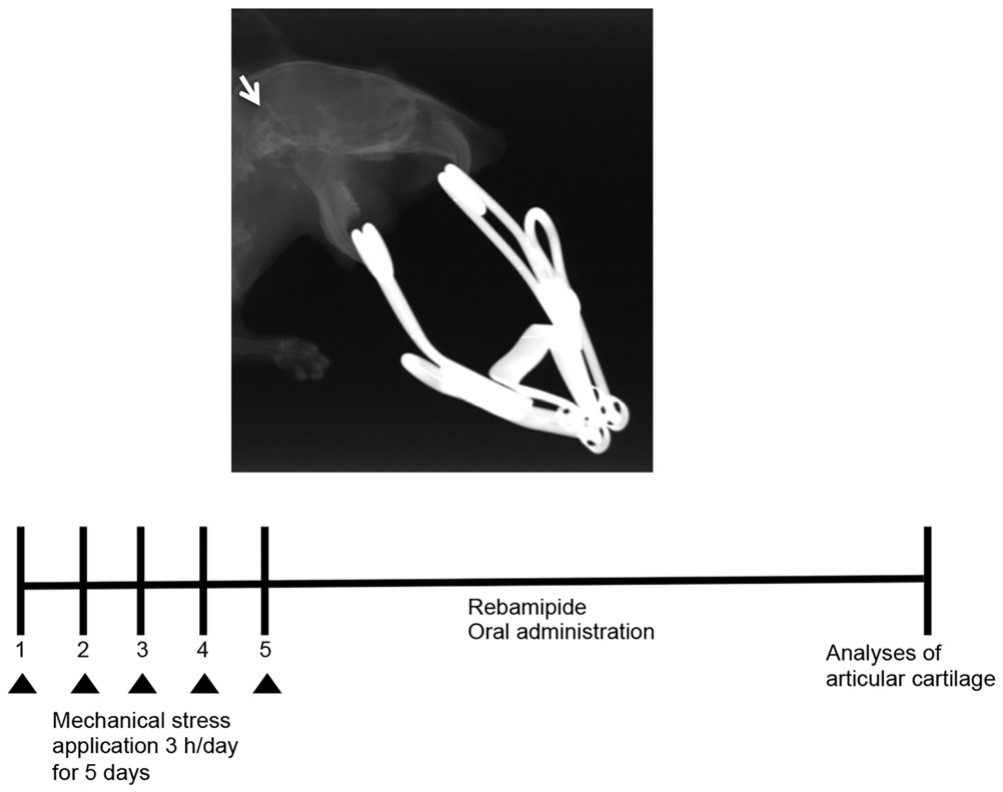
Establishing a TMJ-OA model. The TMJs of C57BL/6 WT mice were subjected to jaw-opening devices that were applied to the interincisal teeth to hold the mandible in the maximal opened position [30]. The mechanical stress was applied for 3 h per day for 5 d while the mice were under general anesthesia that was applied with an intraperitoneal injection of 50 mg/kg somnopentyl.

Following an intraperitoneal injection of 50 mg/kg somnopentyl, adverse mechanical stress was applied to the temporomandibular joint (TMJ) of mice with a consistent and repetitive mouth-opening protocol. A custom-made spring was used to deliver a force of 2 N at maximal mouth opening (measured to be 14 mm, passively, in 8-week-old C57BL/6 WT mice). The TMJ of the mice in the loaded group was subjected to mechanical loading by forceful opening of the mouth for 3 h/d for 5 d (Fig 1). Individual spring forces were measured with a mechanical test system (autograph AG-X 1 kN, SHIMADZU, Kyoto, Japan).

Upon establishment of the TMJ-OA model, TMJ-OA mice were divided among three groups: 0.6 mg/kg rebamipide (R-0.6), 6 mg/kg rebamipide (R-6), and vehicle control (vehicle). Mice in the rebamipide treatment groups received rebamipide (Otsuka Pharmaceutical Company, Tokyo, Japan) dissolved in 0.5% carboxymethylcellulose (CMC) solution (Wako Pure Chemical, Osaka, Japan) for 4 wks. Mice in the vehicle group received CMC alone for 4 wks. Rebamipide in CMC or CMC alone was administered by oral gavage daily after TMJ-OA induction. A fourth group of mice included C57BL/6 WT that were not subjected to mechanical stress.

### Micro-computed Tomography (Micro-CT)

Murine mandibles were resected from each experimental group. The mandibles were free of soft tissues and were fixed overnight in 70% ethanol. The bones were then analyzed by high resolution micro-CT (SkyScan 1176 scanner and associated analysis software, Bruker, Billerica, MA, USA). Briefly, image acquisition was performed at 50 kV and 200 μA. To prevent movement and dehydration of the samples during image acquisition, a plastic wrap was tightly applied. Then, to identify the bone image from the background, thresholding was performed. To achieve 3D images, we used the 3D Creator software (included with the micro-CT scanner) to convert the two-dimensional (2D) images obtained. Each micro-CT image had a resolution of 9 μm per pixel. The microstructural parameters analyzed included the bone volume to trabecular volume ratio (BV/TV), trabecular thickness (Tb.Th), and trabecular separation (Tb.Sp).

### Tissue preparation and histological staining

TMJ tissues were removed and fixed in 4% freshly prepared paraformaldehyde with ethylenediaminetetraacetic acid (EDTA) in PBS for 20 d. Using a microtome (Carl Zeiss HM360, Jena, Germany), serial sagittal sections were cut from paraffin-embedded TMJ tissue blocks. Serial sections of each condyle were stained with hematoxylin-eosin (HE) for histological assessment, and then were stained and counterstained with 0.02% Fast Green to detect proteins and with 0.1% Safranin O to detect cartilage. Condyle sections were also stained with toluidine blue to visualize proteoglycans. Tartrate-resistant acid phosphatase (TRAP) staining was used to identify osteoclasts according to the manufacturer’s instructions (Sigma 387-A, St. Louis, MO, USA). TdT-mediated dUTP-digoxigenin nick-end labeling (TUNEL) staining was performed using an Apoptosis In Situ Detection Kit (Wako Pure Chemical), according to the manufacturer’s directions.

### Immunohistochemistry

Following section deparaffinization and blocking sections were incubated with primary rabbit anti mouse polyclonal antibodies recognizing MMP-13 (Abcam, Cambridge, UK), iNOS (Abcam), or cleaved caspase-3 (Cell Signaling Technology, Danvers, MA, USA) diluted in PBS/0.1% bovine serum albumin overnight at 4°C. The sections were then washed in PBS and incubated with corresponding secondary antibodies at RT for 1h. Bound antibodies were visualized by reaction with 3.3-diaminobenzidine (2.5 mg/mL), and the cells were counterstained with Mayer’s hematoxylin. The stained sections were mounted and analyzed under a BioRevo BZ-9000 microscope (KEYENCE).

### ATDC5 chondroprogenitor cells

ATDC5 mouse chondroprogenitor cells (RIKEN BioResource Center Cell Bank, Tsukuba, Japan) were cultured as a monolayer in DMEM with 10% fetal bovine serum (FBS). Cells were then plated in 24-well tissue culture plates, and 24 h later, the medium was replaced with serum-free DMEM. After an additional 24 h, the cells were pretreated with rebamipide for 2 h and then stimulated with or without 10 ng/ml recombinant human IL-1β (R&D Systems, Minneapolis, MN, USA) for 48 h.

### Detection of mRNA levels

ATDC5 cells were treated with rebamipide and then total RNA was extracted with Nucleo Spin RNA II kits (Macherey-Nagel, Duren, Germany). To estimate RNA concentrations, a NanoDropND-2000 instrument (Nano Drop Technologies, Wilmington, DE, USA) determined absorbance values at 260 nm and 280 nm. When the ratio of these values were < 1.8, samples were not used. To obtain cDNA, total RNA (1 μg) was subjected to a High Capacity RNA to c-DNA Kit (Applied Biosystems, Foster City, CA, USA). Each PCR sample included 10 ng cDNA, 10 μL PowerSYBR Green PCR Master Mix (Applied Biosystems), and 50 μM primers. The primers used were based on mouse sequences and included: MMP-13, 5’-GAT GAC CTG TCT GAG GAA GAC C-3’ (sense) and 5’-GCA TTT CTC GGA GCC TGT CAA C-3’ (antisense); Alpl, 5’-AAC CCA GAC ACA AGC ATT CC-3’ (sense) and 5’-GCC TTT GAG GTT TTT GGT CA-3’ (antisense); osteocalcin, 5’-CAG CGG CCC TGA GTC TGA-3’ (sense) and 5’-GCC GGA GTC TGT TCA CTA CCT TA-3’ (antisense); Col1a15’-GAG CGG AGA GTA CTG GAT CG-3’ (sense) and 5’-GTT AGG GCT GAT GTA CCA GT-3’ (antisense); GAPDH,5’-AGG TCG GTG TGA ACG GAT TTG-3’ (sense) and 5’-TGT AGA CCA TGT AGT TGA GGT CA-3’ (antisense).

Real-time RT-PCR was used to detected *MMP-13* mRNA levels (7500 Real-Time PCR system, Applied Biosystems). The data were subjected to the comparative cycle threshold method (ΔΔCt) and normalized to glyceraldehyde 3-phosphate dehydrogenase (*GAPDH*) levels.

### Macrophage isolation and osteoclast culture

Isolated bone marrow macrophage (BMM) were differentiated into mature multinucleated osteoclasts as described previously [23]. After 6 d of being cultured in macrophage colony-stimulating factor (M-CSF, 20 ng/ml) and receptor activator of nuclear factor kappa-B ligand (RANKL, 100 ng/ml), the cells were stained for TRAP activity (kit 387-A; Sigma).

### Cell viability assay

Cell viability was measured with a water-soluble tetrazolium salt (WST)-8 reagent (Cell Count Reagent SF; Nacalai tesque, Kyoto, Japan) assay. Briefly, ATDC5 cells and BMM cells were each seeded on 96-well plates and cultured as described above for 24 h. The medium was then replaced with medium containing rebamipide at various concentrations, and WST-8 reagent was added to the cultures 48 h later. After incubating for an additional 4 h, absorbance at 450 nm was measured with a microplate reader (SH-1000Lab; Hitachi High-Technologies, Tokyo, Japan).

### Actin ring staining and bone resorption assay

Osteoclasts were generated on bone slices following exposure to 100 ng/ml RANKL and 20 ng/ml M-CSF for 6 d. Actin rings and resorption lacuna were stained as described previously [24,25]. Briefly, cells were fixed in 4% paraformaldehyde and then permeabilized in 0.1% Triton X-100. After being rinsed in PBS, the cells were subsequently immunolabeled with Alexa Fluor 488-phalloidin (Invitrogen, Carlsbad, CA, USA). For the bone resorption assay, osteoclasts were removed and were incubated with 20 μg/ml peroxidase-conjugated wheat germ agglutinin. Visualization of the resorption pits was achieved with 3,3’-diaminobenzidine staining (Sigma).

### Collagen type 1 fragment (Ctx-1) assay

Isolated BMMs were cultured on plastic for 3 d with M-CSF and RANKL, then lifted and re-plated in equal numbers on dentin for 24 h in the presence of osteoclastogenic medium (RANKL and M-CSF with 500 or 1000 nM rebamipide). Bone resorption was analyzed by measuring the release of collagen type 1 into the media. Ctx-1 activity was measured by ELISA (Immunodiagnostic Systems Limited, Boldon, UK).

### Immunoblot analysis

To detect the phosphorylation of IκBα, JNK, ERK, and p38, BMMs were serum-starved for 12 h with or without rebamipide. The cells were subsequently treated with RANKL (100 ng/ml) for 30 min. Cell extracts were lysed in a buffer containing NaCl (150 mM), Tris-HCl (10 mM, pH 7.4), EDTA (5 mM), aprotinin (10 mg/ml), 1% sodium dodecyl sulfate (SDS), leupeptin (50 mg/ml), and phenylmethanesulfonyl fluoride (1 mM) and then were centrifuged. The total protein concentration for each supernatant was determined (BCA Protein Assay, Thermo Fisher Scientific, Rockford, IL, USA) and equal amounts of protein from each sample were individually combined with 2× Laemmli buffer to be separated by 8–12% SDS-PAGE. These proteins were then transferred to polyvinylidene difluoride membranes and blocked with 0.1% Tween 20-TBS (TBS-T) and 5% skim milk. After 1 h at RT, primary rabbit polyclonal antibodies recognizing integrin β_3_ (Cell Signaling Technology) or mouse monoclonal antibodies recognizing NFATc1 (Santa Cruz Biotechnology, Dallas, TX, USA), c-Src, or cathepsin K (Abcam) (all diluted 1:1000) were added as appropriate. After an overnight incubation at 4°C, levels of β-actin were detected using a mouse monoclonal antibody (Sigma-Aldrich) diluted in TBS-T (1:5000) as a loading control. After the membranes were washed with TBS-T (15 min, 3×), the membranes were exposed to secondary horseradish-conjugated anti-rabbit (Cell Signaling Technology) or anti-mouse (Millipore, Billerica, MA, USA) antibodies for 1 h at RT. To detect protein bands, the LumiGLO Western Blot Detection System was applied (Cell Signaling Technology).

### Detection of osteoblast differentiation and mineralization

Bone marrow stromal cells were grown in osteogenic medium containing 20 mM β-glycerophosphate, 50 mM ascorbic acid, and 1 μM rebamipide for 3 wks. Then, the cells were fixed with 70% ice-cold ethanol for 1 h, followed by staining with 0.2% alizarin red S at RT. After 30 min, the cells were destained, left to air dry, and then examined by light microscopy (KEYENCE). Messenger RNA levels of *osteocalcin*, *Alpl*, and *Col1a1* were detected with real-time reverse transcription (RT)-PCR.

### Calcein double labeling

Osteoblast activity was assessed in calcein-labeled, non-decalcified, methacrylamide-embedded sections. Analysis was performed under a KEYENCE microscope (KEYENCE, Osaka, JAPAN) fitted with a 20X objective lens. Quantitative histological parameters were assessed in Bioquant Osteo software (Bioquant Image Analysis Corporation, Nashville, TN, USA).

### Statistical analysis

Data are presented as the mean ± standard deviation (SD). Each sample was analyzed in in triplicate. In addition, each experiment was repeated independently at least two or three other times. Data were statistically analyzed with Student’s *t*-test or one-way analysis of variance (ANOVA) with post-hoc Tukey’s honest significant differences test, as appropriate. A *P*-value less than 0.05 was considered statistically significant.

## Results

### Establishment of a murine model of temporomandibular disorder

**Fig 2:**
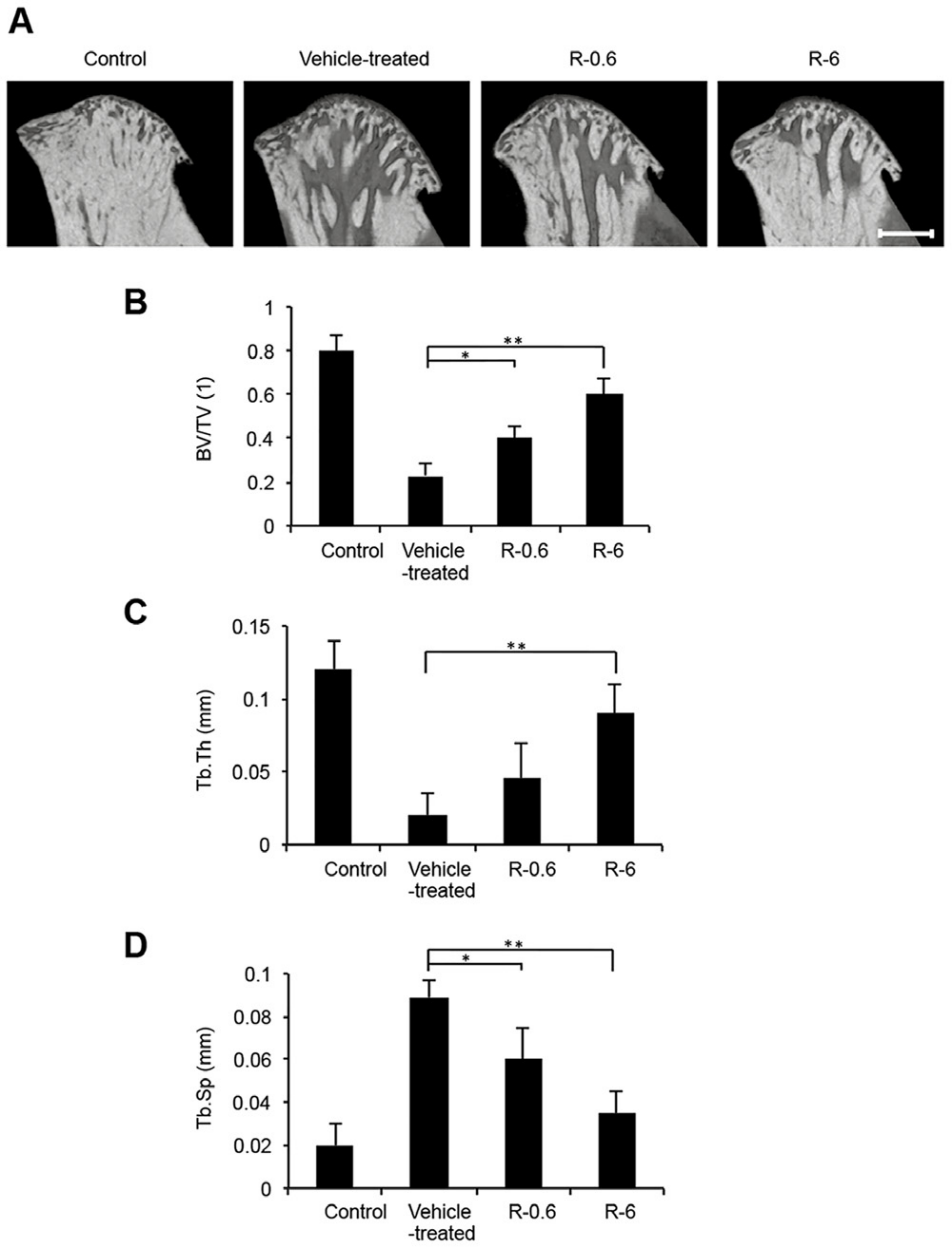
Micro-CT analysis of the mandibular condylar head from rebamipide-treated TMJ-OA mice. A, Based on a 3D reconstruction section of mandibular condyles from rebamipide-treated mice, representative sagittal views from micro-CT scans of the condyles are shown. Scale bar = 500 μm. B, Trabecular BV was determined in representative sagittal plane sections, and these values are presented as BV/TV ratios. C, Tb.Th, trabecular thickness; D, Tb.Sp, trabecular separation. The data presented are the mean ± SD (n = 5). *P < 0.05. **P < 0.01.

**Fig 3:**
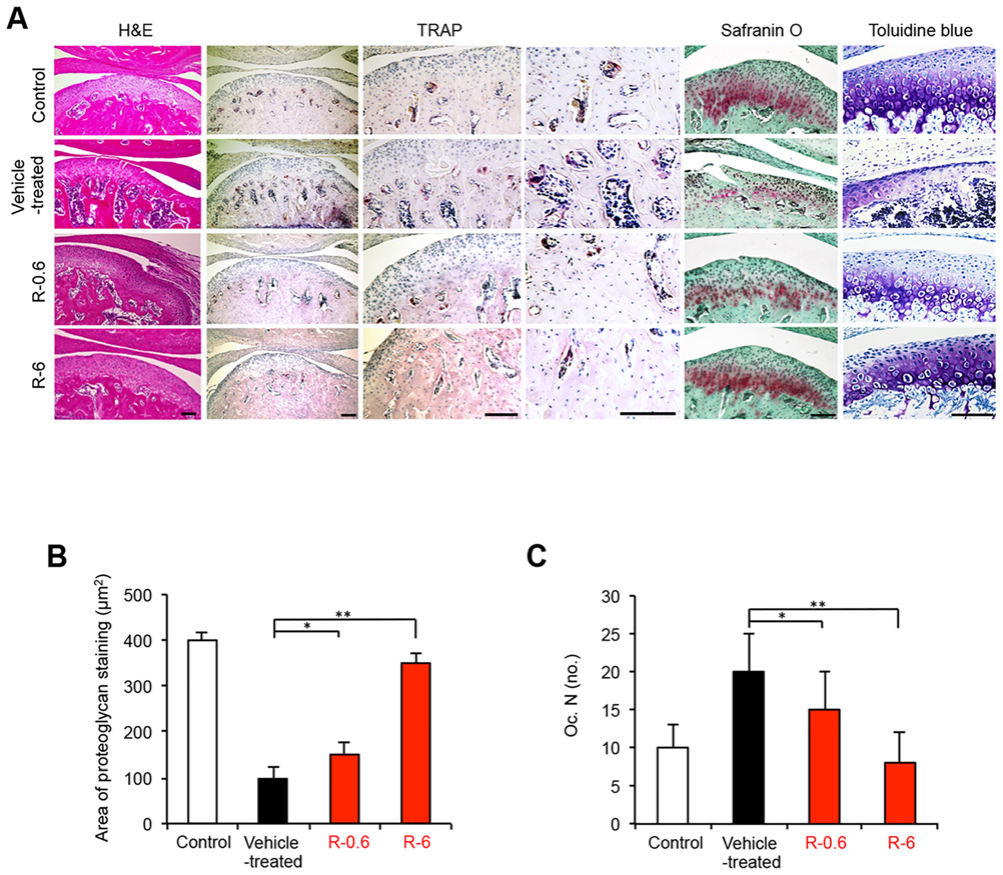
Treatment with rebamipide suppresses mandibular condylar lesions. A, Histologic features of the condylar cartilage obtained from control mice and each of the three experimental TMJ-OA mouse groups (vehicle-treated, R-0.6, and R-6) were observed following the staining of tissue sections from the mandibular condyle with HE, TRAP, Safranin O-fast green, and toluidine blue. Decreased numbers of TRAP-positive osteoclasts, yet no depletion of proteoglycans, were observed in the subchondral bone tissues that were obtained from the R-0.6 and R-6 mice. Extensive cartilage degradation and bone destruction were observed in the tissues obtained from the vehicle-treated group. Rebamipide treatment also preserved the cartilage structure and decreased the depth and the extent of cartilage damage. Scale bar = 100 μm. B, The area (μm^2^) that was stained for proteoglycans in the mandibular condylar cartilage tissues obtained from the four experimental groups of TMJ-OA mice are presented are the mean ± SD (n = 5 mice per group). *P < 0.05; **P < 0.01. C, The number of TRAP-positive cells per mm bone perimeter in the subchondral bone [Oc.N. (no.)] of the condyle tissues obtained from the four experimental groups of TMJ-OA mice are presented are the mean ± SD (n = 5 mice per group). *P < 0.05; **P < 0.01.

A mouse model of TMJ-OA was developed by subjecting the temporomandibular joints (TMJs) of C57BL/6 WT mice to mechanical stress with jaw-opening devices that were applied to the interincisal teeth to hold the mandible in the maximal opened position [26,27] (Fig 1). The mechanical stress was applied for 3 h per day for 5 days. The TMJs were repetitively overloaded and rested. The micro-CT results showed that the BV/TV ratio and the Tb.Th were reduced among different regions of the condylar subchondral bone in the TMJ-OA mice compared with the control mice (Fig 2A–2C). In contrast, the Tb.Sp was significantly greater in the TMJ-OA mice than in the control mice (Fig 2D) When TMJ sections from control WT mice were stained with HE, the articular cartilage exhibited a smooth surface and normal cellularity. In addition, strongly positive staining with Safranin O-fast green and toluidine blue were observed. In contrast, staining of the joints from the TMJ-OA mice with the same three stains revealed OA-like degenerated lesions, including irregularities of chondrocyte alignment in the condylar cartilage layers and subchondral bone loss. Marked depletion of proteoglycans was also observed. Thus, in the experimental mouse model that was established, the early phase of TMJ-OA appears to have been induced (Fig 3).

### Rebamipide attenuates cartilage degeneration in TMJ-OA model in a dose-dependent manner

Rebamipide dissolved in CMC, or CMC alone, was administered orally each day after the TMJ-OA model was established (Fig 1). Two doses of rebamipide were applied, 0.6 mg/kg (R-0.6) and 6 mg/kg (R-6). The micro-CT results showed that the BV/TV and the Tb.Th were increased in several regions of the condylar subchondral bone in the rebamipide-treated mice compared with the TMJ-OA mice (Fig 2A–2C). In contrast, the Tb.Sp was significantly smaller in the rebamipide-treated mice than in the TMJ-OA mice (Fig 2D). After rebamipide or vehicle alone were administered daily for 4 wks, cartilage from the control mice and from each of the three experimental TMJ-OA mouse groups (vehicle-treated, R-0.6, and R-6) were also assessed with Safranin O and toluidine blue staining (Fig 3A). The TMJ joints of the mice treated with rebamipide exhibited a significant and dose-dependent reduction in cartilage compared with the TMJ joints of vehicle-treated mice. Cartilage thickness and degree of proteoglycan content in R-6 mice did not differ from those of the control mice (Fig 3A and 3B).

### Rebamipide effects on osteoclast activity in condyle subchondral bone

TRAP staining was used to examine the effects of rebamipide on osteoclastogenic activity *in vivo* (Fig 3A). The number of TRAP-positive osteoclasts that were counted in the condyle subchondral bone was considered a readout of osteoclast activity. For the samples analyzed from the control mice and the three experimental TMJ-OA mouse groups, the number of TRAP-positive osteoclasts was the lowest in the R-6 group compared with the vehicle-treated group, thereby indicating that osteoclast activity was significantly attenuated with rebamipide treatment (Fig 3A and 3C).

### Rebamipide effects on the apoptosis of mandibular condylar cartilage cells

**Fig 4:**
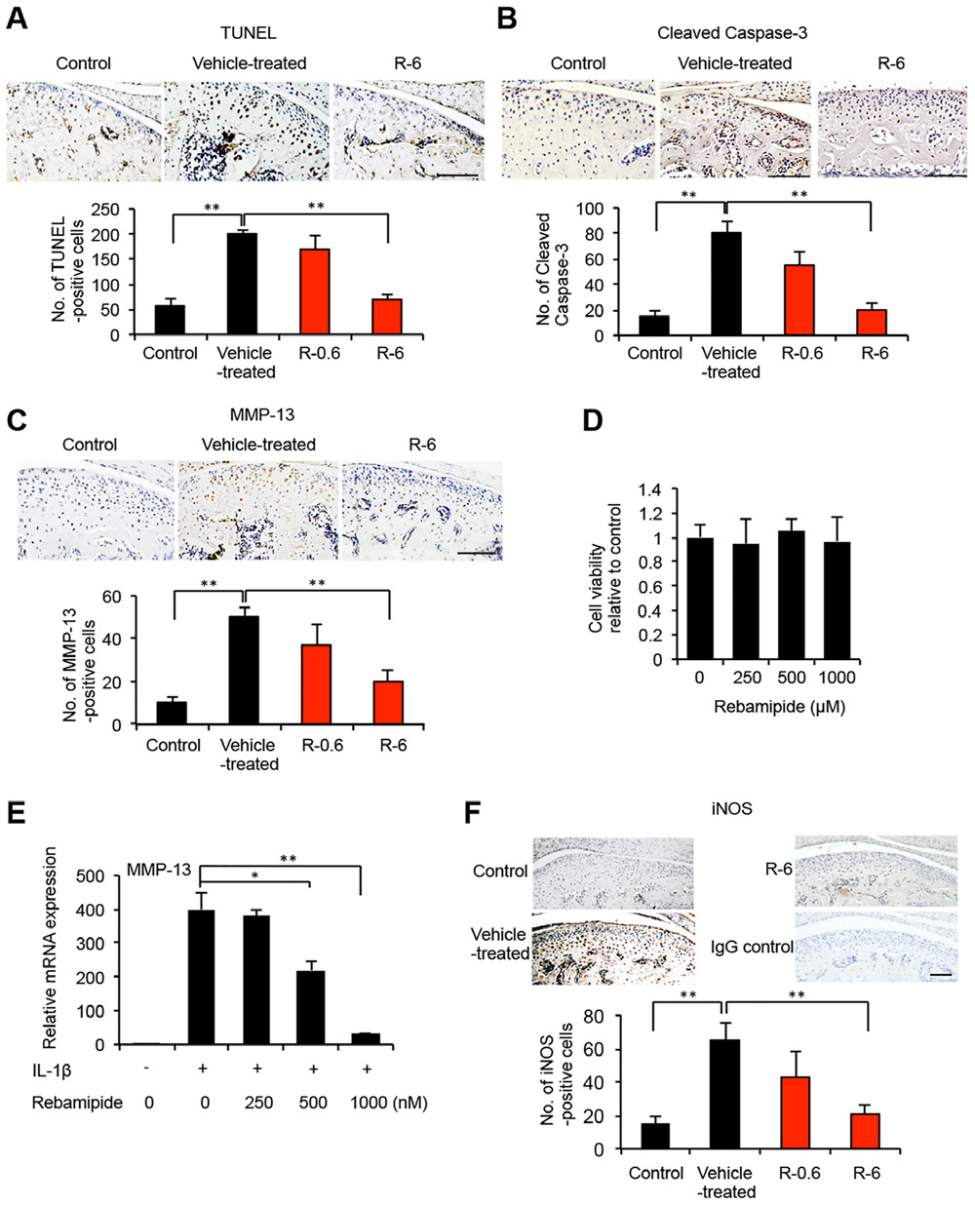
Effects of rebamipide on apoptosis, MMP-13, and iNOS for the mandibular chondrocyte cells in the mouse model of TMJ-OA. A, Representative tissue sections from the mandibular condyle of the three experimental groups of TMJ-OA mice (control, vehicle-treated, and R-6; n = 5 mice/group) that underwent TUNEL staining. The number of TUNEL-positive cells (stained brown) for the vehicle-treated, R-0.6, and R-6 tissues were determined, and the data are presented as the mean ± SD. The number of TUNEL-positive cells was significantly attenuated in the condylar cartilage tissues of the R-6 mice compared with the vehicle-treated mice. **P < 0.01. Scale bar = 100 μm. B, C, Serial sections of condylar cartilage from the vehicle-treated and R-6 tissues stained in A were immunostained for cleaved caspase-3 (B) and MMP-13 (C). Expression of both targets were dramatically attenuated in the condylar cartilage of the R-6 mice compared with the vehicle-treated mice. **P < 0.01. Scale bar = 100 μm. D, ATDC5 cells were treated with various concentrations of rebamipide for 48 h, and cell viability was measured in WST-8 assays. E, ATDC5 cells were cultured with or without IL-1β in the absence or presence of rebamipide (Reba) at various concentrations as indicated for 48 h following an initial 24 h of serum starvation. The levels of *MMP-13* mRNA were measured by quantitative real-time PCR. Detection of *GAPDH* was used as an internal control. Ct cycles of *MMP-13* were in the range of 22.0–26.0. Ct cycles of *GAPDH* were in the range of 15.0–15.7. The data presented are the mean ± SD for three independent experiments that were performed per group. *P < 0.05; **P < 0.01. F, Serial sections of condylar cartilage tissues from vehicle-treated and R-6 mice were immunolabeled for iNOS expression. A lower number of iNOS-positive cells were observed in R-6 than in vehicle-treated tissues. **P < 0.01. Scale bar = 100 μm. As a negative control, mandibular articular cartilage obtained from R-6 mice were stained with rabbit IgG (isotype control).

Recent studies have suggested that cell death in OA cartilage occurs primarily via apoptosis [28,29]. Thus, TUNEL assays were performed to determine whether abnormal chondrocyte apoptosis preferentially occurred in degraded cartilage. A significant decrease in the number of TUNEL-positive apoptotic chondrocyte cells was observed in the mandibular condyle of the R-6 mice compared with the vehicle-treated mice (P < 0.01; Fig 4A).

Detection of cleaved caspase-3 was also used to distinguish apoptotic chondrocytes from cells that died by other mechanisms, such as necrosis [28]. In the mandibular condylar cartilage obtained from the control mice, cells positive for cleaved caspase-3 were observed to be progressively distributed within whole layers of the cartilage (Fig 4B). In contrast, significantly lower levels of cleaved caspase-3 were detected in the condylar cartilage tissues from the R-6 mice (P < 0.01; Fig 4B). Taken together, these results suggest that rebamipide contributes to the apoptosis of mandibular condylar cartilage by affecting the signaling that is mediated by activated caspases.

### Rebamipide effects on the expression levels of MMP-13 in the condylar cartilage of TMJ-OA mice

Degenerative changes in the cartilage matrix may be due to reduced matrix synthesis, increased matrix degradation, or both. To distinguish these possibilities, expression levels of MMP-13 were examined. In the mandibular condylar cartilage that was obtained from the vehicle-treated TMJ-OA mice, MMP-13-positive cells were progressively distributed (Fig 4C). However, in the R-6 mice, fewer MMP-13-positive chondrocytes were observed in the mandibular condyle compared with the vehicle-treated mice (Fig 4C).

### Rebamipide effects on MMP-13 gene expression in ATDC5 chondroprogenitor cells

To more precisely examine the effects of rebamipide on the function of chondrocytes, gene expression of *MMP-13* was detected in the mouse embryonal carcinoma-derived cell line, ATDC5, which represents chondroprogenitor cells. WST-8 cell viability assays revealed no cytotoxic effects of 48-h rebamipide exposure on ATDC5 cells, compared to untreated control cells (Fig 4D). The ATDC5 cells were treated with or without IL-1β, a molecule known to be a key factor in the induction of MMP-13 synthesis in chondrocytes [30]. Gene expression of *MMP-13* increased after IL-1β was added to, andthe ATDC5 cells, and this effect was reduced when the cells were treated with 1000 nM rebamipide (Fig 4E). These data support the *in vivo* finding that rebamipide potentially contributes to the maintenance of condylar cartilage via MMP-13.

### Reduced expression of iNOS in mandibular condylar cartilage from rebamipide-treated TMJ-OA mice

NO inhibits the synthesis of proteoglycan and collagen II in chondrocytes, and in mouse models of OA that are depleted of iNOS, less cartilage degradation has been observed compared with WT littermates [31,32]. To determine the degree of oxidative damage that the condylar cartilage of rebamipide-treated TMJ-OA mice undergo, immunohistochemistry assays were performed to assess iNOS expression after four weeks of oral administration of rebamipide. The expression of iNOS markedly increased in the articular cartilage of the TMJ joints of the vehicle-treated mice, while the expression of iNOS was markedly reduced in the joints of the R-6 mice (Fig 4E).

### Rebamipide inhibits osteoclast differentiation in a dose-dependent manner

**Fig 5:**
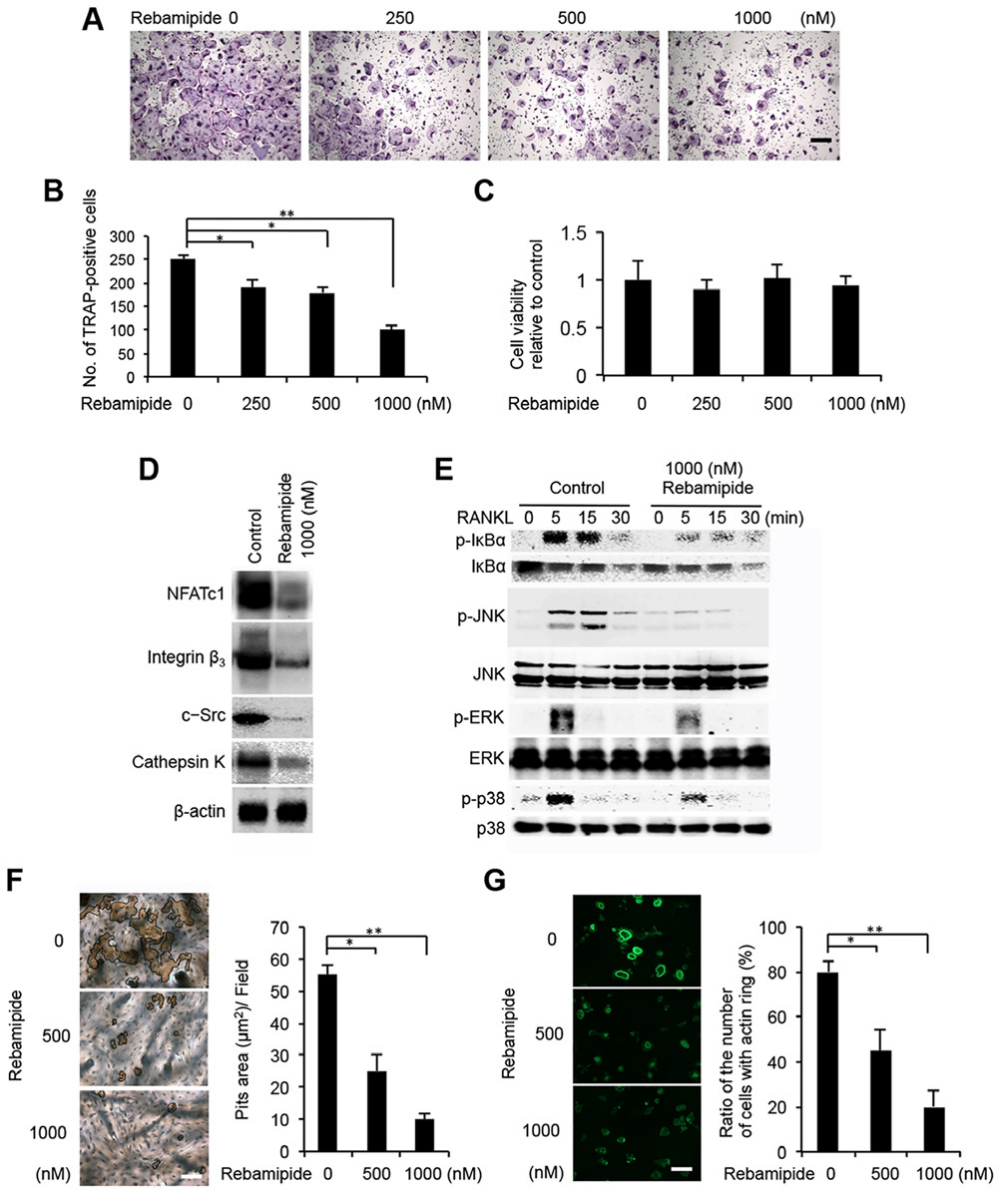
Rebamipide inhibits RANKL-mediated osteoclastogenesis. A, Representative images of BMM that were cultured in the presence of rebamipide at the indicated concentrations during osteoclast differentiation. The cells were stained with TRAP. Scale bar = 100 μm. B, The number of TRAP-positive mature osteoclasts that were detected in the cells described in Fig 5A. Data are presented as the mean ± SD of three independent experiments. **P < 0.01. C, BMMs were treated with various concentrations of rebamipide for 48 h, and cell viability was measured by WST-8 assay. D, Expression levels of NFATc1, integrin β_3_, c-Src, and cathepsin K that were detected in western blots of lysates collected from 1000 nM rebamipide-treated BMM versus in untreated BMM (control) 3 d after RANKL stimulation. Detection of β-actin was used as a loading control. E, BMM that were serum- and cytokine-starved for 12 h with or without 1000 nM rebamipide were exposed to RANKL (100 ng/mL) for the indicated periods of time. Levels of phosphorylated (p-) and unphosphorylated IκBα, JNK, ERK, and p38 were detected by immunoblot. The unphosphorylated forms of the proteins served as loading controls. F, Bone resorbing activity of osteoclasts that were treated with rebamipide. Mature osteoclasts were cultured on bone slices and then were treated with rebamipide at the indicated concentrations for 6 d in the presence of 100 ng/ml RANKL and 20 ng/ml M-CSF. The graph indicates the relative amount of the resorbed area at each concentration of rebamipide. Scale bar = 100 μm. *P < 0.05; **P < 0.01. G, Immunofluorescence detection of actin in osteoclasts that were treated with or without rebamipide (1000 nM). Scale bar: 100 μm. The ratio of the number of cells with an actin ring is reported in the accompanying bar graph. Scale bar = 100 μm. *P < 0.05; **P < 0.01. H, Collagen type 1 fragment release from pre-osteoclasts seeded in equal number on dentin for 24 h in the presence of osteoclastogenic medium with RANKL and M-CSF alone or supplemented with 1000 nM rebamipide.

To confirm that BMM to osteoclast differentiation is sensitive to rebamipide, BMMs were treated with rebamipide (0–1000 nM) for 5 d with RANKL (100 ng/ml) and M-CSF (20 ng/ml). Rebamipide reduced the generation of TRAP-positive osteoclasts in a dose-dependent manner (Fig 5A). Furthermore, when cells were pretreated with 1000 nM rebamipide, the number of osteoclasts were 40% less than cells incubated with RANKL and M-CSF (Fig 5B). WST-8 cell viability assays revealed no cytotoxic effects of 48-h rebamipide exposure on BMMs, compared to untreated control cells (Fig 5C).

### Rebamipide suppresses osteoclast gene expression

Osteoclasts are derived from monocyte-macrophage lineages. Moreover, the terminal differentiation of osteoclasts has been accompanied by the expression of transcription factor, NFATc1, as well as integrin β_3_, c-Src, cathepsin K, and other markers of osteoclast differentiation [33]. In the western blot analysis of lysates collected from 1000 nM rebamipide-treated BMMs 3 d after RANKL stimulation versus untreated BMMs, lower levels of NFATc1, integrin β3, c-Src, and cathepsin K were detected (Fig 5D). These results suggest that rebamipide blocks osteoclast differentiation by inhibiting NFATc1 expression, and this affects the downstream expression of osteoclast-related genes.

Next, signaling events stimulated by rebamipide in response to RANKL were examined. Activation of NF-κB is crucial for RANKL-induced osteoclastogenesis [33], and in the cytosol, NF-κB is bound to IκBα and is inactive. However, upon degradation of IκBα, NF-κB is released and becomes active [33]. Therefore, it was investigated whether rebamipide inhibits the phosphorylation and degradation of IκBα. Accordingly, BMMs were pretreated for 8 h with 1000 nM rebamipide, and then protein levels of IκBα were determined after an additional 30 min of exposure to RANKL (100 ng/ml). It was observed that rebamipide significantly suppressed RANKL-induced phosphorylation of IκBα (Fig 5E).

In addition to the NF-κB signaling pathway, activation of the MAPK pathway also plays a pivotal role in osteoclastogenesis [33]. To evaluate the effects of rebamipide on MAPK signaling following the stimulation of RANKL in BMMs, Western blot analysis was used to examine phosphorylation of JNK, ERK, and p38. Rebamipide was found to significantly inhibit RANKL-induced phosphorylation of all three targets, while the levels of total JNK, ERK, and p38 were unaffected by RANKL and rebamipide treatments (Fig 5E). These results indicate that rebamipide can inhibit RANKL-induced activation of NF-κB and MAPK signaling in osteoclasts.

### Rebamipide inhibits the bone-resorbing activity of osteoclasts by disrupting actin rings

Cytoskeletal reorganization, such as actin ring formation, is important for the bone-resorbing function of mature osteoclasts [34]. RANKL-induced pit formation assays revealed that rebamipide treatment inhibits the bone-resorbing activity of osteoclasts partially at 500 nM, and almost completely at 1000 nM, as indexed by the release of type 1 collagen fragments (Ctx-1) into the medium (Fig 5F and S1A Fig). Consistent with these observations, the actin ring disappeared essentially within 8 h of rebamipide treatment (Fig 5G), suggesting that rebamipide suppression of bone resorbing activity may be due to disruption of actin rings. To determine whether rebamipide affects mature resorptive cell activity, we plated the same number of osteoclast precursors (cells that have been in culture with RANKL and M-CSF for 3 d) on dentin for 24 h. In this circumstance, in which an equal number of TRAP- positive cells were present on each dentin slice, the quantities of collagen fragments mobilized did not differ between osteoclastogenic medium with RANKL and M-CSF alone versus medium supplemented with 1000 nM rebamipide (S1B Fig). Delivered with intact cytoskeletal organization, rebamipide reduces osteoclast differentiation, but does not alter the resorptive capacity of mature osteoclasts.

### Osteoblastogenesis in bone marrow stromal cells is not affected by rebamipide

**Fig 6:**
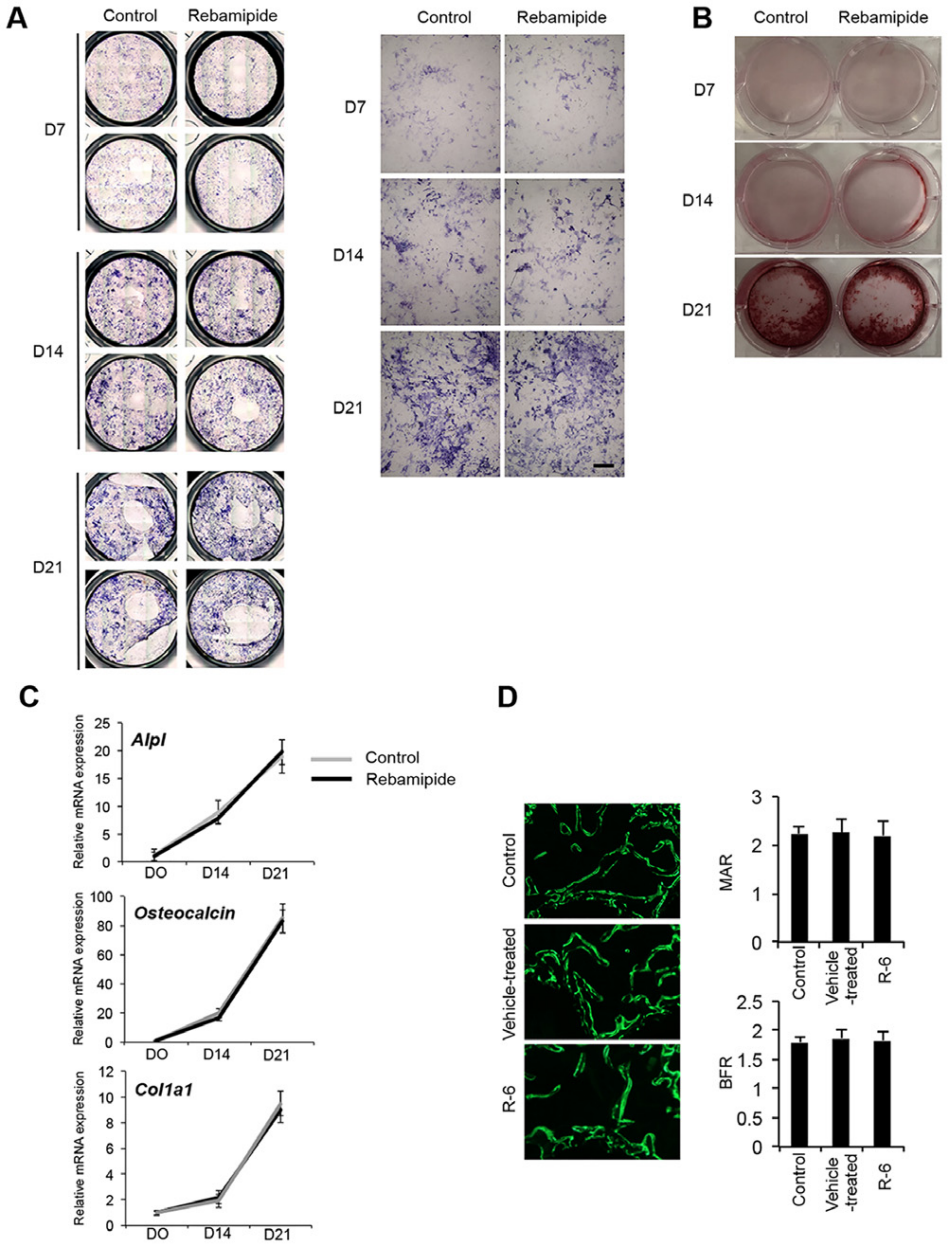
Effects of rebamipide on osteoblastogenesis. A, B, Osteoblastic cells were cultured from the bone marrow stromal cells of C57BL/6 WT mice, and the cells were subsequently cultured with or without 1000 nM rebamipide for up to 21 d. Parallel cultures of the cells were stained with ALP (A) and Alizarin Red (B) after 7, 14, and 21 d of culturing. Scale bar = 100 μm. C, Total RNA was isolated from osteoblastic cells that were cultured with (black line) or without (grey line) rebamipide (1000 nM). Real-time PCR was used to analyze the relative expression levels of the osteoblast-related marker mRNAs, *Alpl*, *osteocalcin*, and *Col1a1*, after 0, 14, and 21 d of culturing. Data are expressed as the copy numbers of these markers normalized to *GAPDH* expression ± SD. Ct cycles of *Alpl*, *osteocalcin*, *Col1a1*, *GAPDH* were in the range of 19.8–21.5, 19.5–22.9, 20.2–22.8, and 14.7–15.7, respectively. D, Fluorescent images of newly formed bones in control, TMJ-OA and R-6 mice injected with calcein on days 0 and 5 and sacrificed on day 7.

To determine the effect of rebamipide on the formation of osteoblasts that can be generated from bone marrow stromal cells, an *in vitro* culture system was established. Analysis of alkaline phosphatase (ALP) and alizarin red staining showed no effects of rebamipide treatment on osteoblast formation (Fig 6A and 6B). In addition, temporal mRNA expression profiles of the osteoblastic markers, *Alpl*, *osteocalcin*, and *Col1a1*, were indistinguishable between osteoblastic cells that were cultured with or without 1000 nM rebamipide (Fig 6C). Our observations in an *in vitro* culture system established in the absence of osteoblasts suggest that rebamipide prevented osteoclast formation by affecting osteoclast precursor cells directly. This supposition is supported by our *in vivo* analysis of bone mineral apposition shown with calcein double-labeling (Fig 6D), which revealed no significant difference in bone formation rates (BFRs) and mineral apposition rates (MARs) between control, vehicle-treated, and R-6 animals. These results suggest that the increased bone mass observed in R-6 mice was not due to aberrant osteoblast activity.

## Discussion

Rebamipide has been widely applied as a gastroprotective drug against gastritis and gastric ulcers, and has exhibited mucin secretagogue activity, anti-inflammatory actions, and antibacterial effects [35–38]. Interestingly, a recent study showed that adjunct rebamipide therapy is also effective for preventing the occurrence of peptic ulcers in arthritic patients that are taking a COX-2-selective inhibitor [39]. It has been demonstrated that oral administration of rebamipide can reduce the clinical and histologic scores in animal models of rheumatoid arthritis, including collagen-induced arthritis and SKG mice [40,41]. There has been only one recent report regarding the inhibitory effects of rebamipide on pain production and cartilage degeneration in experimentally induced rat knee OA [42]. The hypothesis for the present study was that the anti-inflammatory activity of rebamipide in mandibular condyles would represent a beneficial therapeutic approach for TMJ-OA. To date, the cause-and-effect relationship between abnormalities in the subchondral bone and the development of TMJ-OA has not been established. However, the results of the current study provide valuable insights.

In the present study, the TMJ-OA model that was established was characterized by OA-like degenerated lesions, irregularities in the alignment of chondrocytes in the condylar cartilage layers, subchondral bone loss, and marked depletion of proteoglycans. It was reported previously that forced mouth opening decreases subchondral bone volume in mice [43]. It has also been reported in rabbit and rat model studies that repetitive, steady jaw-opening was effective for developing OA-like changes compatible with the clinical presentation of TMJ-OA patients [26,44]. Furthermore, these results are consistent with previous results reported for early TMJ-OA produced by surgical manipulation of the joint [45], local application of chemicals [3], biomechanical stimulation from abnormal occlusion [4,46,47], and genetic modification [48,49]. For cartilage that is affected by OA, an increase in the number of chondrocytes that undergo cell death has been observed [50]. In addition to cell death, the remaining chondrocytes of cartilage affected by OA have been found to exhibit changes in their synthesis or degradation of the ECM as a result of changes in anabolic and catabolic gene expression [50]. In particular, MMP-13 has been shown to play a role in the resorption of subchondral bone and the degradation of articular cartilage to affect the histological phenotype of OA. However, in the rebamipide-treated TMJ-OA joints, obvious cartilage degradation, manifested as excessive chondrocyte apoptosis and increased expression of MMP13 by chondrocytes, was attenuated in the hypertrophic layer of condylar cartilage in a dose-dependent manner compared with the vehicle-treated TMJ-OA joints. Taken together, oral administration of rebamipide successfully reduced TMJ-OA severity through regulation of MMP-13.

The pathogenesis of OA also involves the continuous exposure of cells and the ECM to oxidative stress. Specifically, elevated production of ROS in combination with the depletion of antioxidants has been implicated in the progression of OA [51], and the resulting imbalance between oxidants and antioxidants is referred to as oxidative stress. It is possible that ROS act at different levels of the cartilage degradation process, and this may include an inhibition of matrix formation and an induction of matrix degradation enzymes [52]. Due to the involvement of increased apoptosis in chondrocytes in OA pathogenesis, ROS are considered a potential treatment target. One well-known marker of oxidative stress is iNOS, and immunohistochemical staining for iNOS after TMJ-OA induction was performed in the present study. All chondrocytes were positive for iNOS expression, except in the cartilage of the rebamipide-treated TMJ-OA mice where expression of iNOS was dramatically attenuated. Thus, oxidative stress in the cartilage of the TMJ-OA joint, as well as the chondroprotective effects of rebamipide, may be associated with the ROS-scavenging property of rebamipide.

Excessive subchondral bone resorption plays a central role in TMJ-OA [4,47,53], while osteoclast activity plays a pivotal role in bone destruction in early stage TMJ-OA. In the present study, increased recruitment of osteoclasts was observed in the subchondral bone regions that composed the areas of cartilage degradation in the TMJ-OA mice group *in vivo*, while the numbers of TRAP-positive osteoclasts were markedly reduced in the condyle of the rebamipide-treated TMJ-OA mice. In this study, we also determined the effect of rebamipide on the formation of osteoclasts from BMMs *in vitro*. Treatment of BMM with rebamipide was found to inhibit RANKL-induced formation of osteoclasts from precursor cells without cytotoxicity.

In the present study, rebamipide treatment was found to reduce RANKL-induced expression of NFATc1, integrin β_3_, c-Src, and cathepsin K. RANKL also activates JNK, ERK, and p38, which have been reported to play important roles in early osteoclastic differentiation [33]. When the effects of rebamipide on the activation of these MAPKs were investigated, phosphorylation of all three kinases was inhibited, thereby indicating a non-specific downregulation of MAPKs. These results are similar to those reported for acteoside, a major anti-inflammatory and antioxidant compound that is derived from Rehmannia glutinosa, an herb that is widely used in traditional Oriental medicine [54]. Thus, phosphorylation of MAPK may contribute to the anti-osteoclastogenic effect mediated by rebamipide in RANKL-stimulated BMMs.

Activation of the NF-κB pathway is a key step in RANKL-induced osteoclast differentiation [33], with activation of NF-κB occurring following the targeting of IκBα for ubiquitin-dependent degradation [33]. In the present study, rebamipide inhibited the cytoplasmic degradation of IκBα, and increased the levels of NF-κB transactivation. Thus, it appears that repabmipide is able to target NF-κB and MAPK signaling, and this negatively affects the formation of osteoclasts from macrophage stimulated with RANKL, as well as osteoclast differentiation.

It has been demonstrated that the formation of new bone requires osteoblasts. Therefore, it is hypothesized that the ability to enhance the differentiation or proliferation of osteoblasts would facilitate bone formation [55]. However, in the present study, when bone marrow stromal cells were exposed to β-glycerophosphate, rebamipide, and osteoblastogenic medium containing α-MEM and ascorbic acid, the mineralization or differentiation of osteoblasts was not affected. Based on these results, rebamipide appears to contribute to an anti-resorption effect, while not directly affecting bone formation. Therefore, bone-specific parameters that are relevant *in vivo* versus *in vitro* need to be investigated to determine if rebamipide provides a beneficial effect on osteoblastogenesis.

In this study, obvious cartilage degradation, manifested as excessive chondrocyte apoptosis and increased expression of MMP-13 by chondrocytes, was attenuated in the hypertrophic layer of condylar cartilage in a dose-dependent manner in the rebamipide-treated TMJ-OA joints compared with the vehicle-treated TMJ-OA joints. Additional studies are needed to better understand how these changes induce chondroprotection and affect the homeostasis of cartilage ECM. It also remains unclear whether rebamipide affects the survival of OA chondrocytes. However, the capacity for rebamipide to mediate highly effective anti-resorptive activity and to suppress osteoclast formation were observed. Thus, rebamipide should continue to be investigated as a potential treatment for patients with TMJ-OA.

## Supporting Information

S1 Fig Collagen type 1 fragment release. A, Resorptive activity was determined by collagen type 1 fragment (CrossLaps) ELISA of culture media treated with 500 or 1000 nM rebamipide for 5 d in the presence of osteoclastogenic medium with RANKL and M-CSF. *P < 0.05; **P < 0.01. B, Collagen type 1 fragment release from pre-osteoclasts, seeded in equal number on dentin for 24 h in the presence of osteoclastogenic medium including RANKL and M-CSF with 500 or 1000 nM rebamipide.
(TIF)
