# Correction: Rebamipide Attenuates Mandibular Condylar Degeneration in a Murine Model of TMJ-OA by Mediating a Chondroprotective Effect and by Downregulating RANKL-Mediated Osteoclastogenesis

**DOI:** 10.1371/journal.pone.0162032

**Published:** 2016-08-25

**Authors:** Takashi Izawa, Hiroki Mori, Takehiro Shinohara, Akiko Mino-Oka, Islamy Rahma Hutami, Akihiko Iwasa, Eiji Tanaka

The third author's name is spelled incorrectly. The correct name is: Takehiro Shinohara.
