# Supplementary figures and images for: Rebamipide Attenuates Mandibular Condylar Degeneration in a Murine Model of TMJ-OA by Mediating a Chondroprotective Effect and by Downregulating RANKL-Mediated Osteoclastogenesis
Source: PLoS One. 2016 Apr 28;11(4):e0154107. doi: 10.1371/journal.pone.0154107 (PMC4849711; doi:10.1371/journal.pone.0154107)

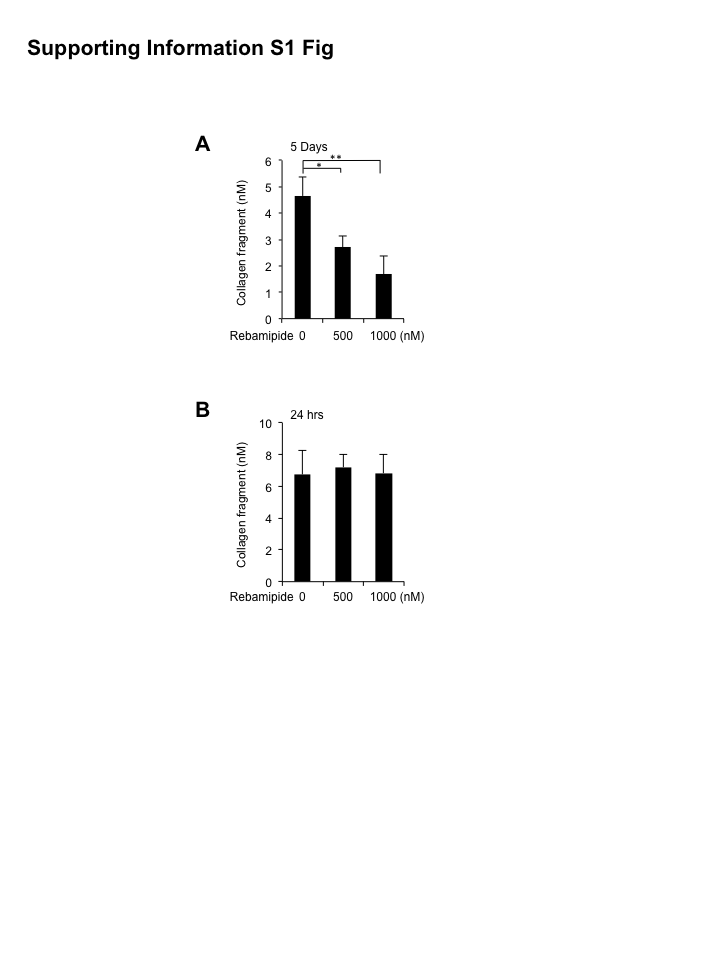

Supplement: S1 Fig — A, Resorptive activity was determined by collagen type 1 fragment (CrossLaps) ELISA of culture media treated with 500 or 1000 nM rebamipide for 5 d in the presence of osteoclastogenic medium with RANKL and M-CSF. *P < 0.05; **P < 0.01. B, Collagen type 1 fragment release from pre-osteoclasts, seeded in equal number on dentin for 24 h in the presence of osteoclastogenic medium including RANKL and M-CSF with 500 or 1000 nM rebamipide. (TIF) [file pone.0154107.s001.tif]
